# A functional SNP of the core promoter region within goat C*DC25A* gene affects litter size

**DOI:** 10.3389/fvets.2024.1471123

**Published:** 2025-02-05

**Authors:** Taiyuan Zhang, Jingxuan Wang, Yangyang Bai, Qian Wang, Ke Wang, Haijing Zhu, Lei Qu, Zhengang Guo, Chuanying Pan, Xianyong Lan

**Affiliations:** ^1^College of Animal Science and Technology, Northwest A&F University, Yangling, China; ^2^Shaanxi Provincial Engineering and Technology Research Center of Cashmere Goats, College of Life Science, Yulin University, Yulin, China; ^3^Testing Center for Livestock and Poultry Germplasm, Guiyang, China

**Keywords:** goat, litter size, CDC25A gene, promoter activity, SNP, KASP

## Abstract

The *Cell division cycle 25A* (*CDC25A*) gene has been considered as a candidate gene associated with reproductive traits for goat breeding. In this study, five truncated fragments divided at position-2285 nt to +198 nt were amplified and cloned into the luciferase reporter vectors to identify the core promoter. The luciferase reporter assay showed that the core promoter of *CDC25A* was located at position-663 nt to-237 nt. Afterwards, a single nucleotide polymorphism (NC_030829.1:g.51731829A > C) at the core promoter was detected using sequencing and KASP in a population of 1,016 goats and luciferase reporter vectors carrying the A allele or C allele were transfected into cells, respectively. The results displayed that the higher relative luciferase activity was observed in plasmids carrying the A allele rather than the C allele. The litter size of individuals with the AA genotype was significantly better than those with other genotypes, which corresponded to increased transcriptional activity in plasmids carrying the A allele. In short, our study provides a potential molecular genetic marker for improving reproductive efficiency in goat breeding.

## 1 Introduction

For thousands of years, goats *(Capra hircus)* have provided humans with meat, milk, and fiber products. In today’s goat industry, improving economic returns through breeding programs remains a key focus. Reproductive traits, as critical phenotypic characteristics, have a significant impact on the economic viability of goat production. However, reproductive traits are quantitative traits with low heritability, and traditional breeding programs have difficulty to improve these traits in a short time. Marker-assisted selection (MAS) is a method based on morphological, biochemical or DNA/RNA makers to select individuals with better phenotypic traits, which is quicker and more effective than traditional breeding programs (1, 2). Using MAS to identify and choose genotypes that enhance fertility can significantly boost reproductive efficiency in goats over a short period.

The *CDC25A* is a dual-specificity phosphatase of the *CDC25* family, which plays a crucial role in the regulation of the eukaryotic cell cycle (3, 4). In reproduction, *CDC25A* plays a crucial role in spermatogenesis, where its decreased expression is linked to infertility and failed sperm retrieval (5). Additionally, overexpression of miR-15b, which targets the 3’-UTR of *CDC25A*, negatively impacts *CDC25A* activity and is vital for proper spermatogenesis (6). In mouse oocytes, downregulation of *CDC25A* leads to the release from metaphase II arrest, demonstrating its importance in maintaining the cell cycle during meiosis (7). Among livestock, the goat *CDC25A* gene shares a closer evolutionary relationship with other species within the *Bovidae* family (Supplementary Figure S1). Dong et al. (8) found that *CDC25A* showed lower expression levels in cattle-yak (*Bos taurus* × *Bos grunniens*) testis than those in cattle (*Bos taurus*) and yak (*Bos grunniens*), which was considered to correlate the infertility in cattle-yak. The gene expression heat map referring to the Ruminant Genome Database^1^ (9) displayed that *CDC25A* showed relatively high expression levels in testis and ovary of goats, which may imply its potential function in reproduction (Supplementary Figure S3). For goat breeding, nucleotide polymorphisms of goat *CDC25A* were considered as potential molecular markers. For instance, an insertion/deletion mutation located in the intron of *CDC25A* has been found to be correlated with goat litter size (10) as well as growth traits (11). In a study by Wang et al. (12), *CDC25A* was identified as a candidate gene underlying strong selection signature with goat body size. Genes in the same family generally have similar structure and functions (13). Wang et al. (2) used whole genome sequencing (WGS) to identify a variant in *CDC25C*, another member of the *CDC25* family, was significantly related to goat litter size. These findings stimulated our interest in exploring the relationship between *CDC25A* and goat reproductive traits.

Most studies in the *CDC25A* gene have been limited to coding region and intron variants that may affect protein function and mRNA splicing, yet polymorphisms within the promoter have been ignored. A promoter is a DNA sequence, which is needed to turn a gene on or off (14). Generally, the promoter region ranges from 2,000 bp upstream to 500 bp downstream of the transcription start site (TSS) in animals (15). The core promoter, including the RNA polymerase binding site and cis-acting elements, etc., plays a crucial role in regulating transcription of the gene (16). Mutations in the core promoter probably alter transcriptional activity and thus affect phenotypic traits. Hence, identifying the core promoter in goat *CDC25A*, scanning the mutation within it, and assessing the correlation of the mutation and reproductive traits are of great significance.

Herein, we identified promoter activity in different truncated fragments of the goat *CDC25A* gene, discovered a novel SNP in the core promoter that affected the transcriptional activity, and analyzed the correlation between the novel SNP and litter size in goat. These findings would provide a potential molecular genetic marker to improve reproductive efficiency in goat breeding.

## 2 Materials and methods

All animal experiments were approved by the Animal Care and Use Committee of Northwest A&F University (protocol No.314020038) and conformed to the animal welfare laws and guidelines in this study.

### 2.1 Samples and data

A total of 1,016 ear samples of adult female Shaanbei White Cashmere (SBWC) were randomly collected in Yulin city, Shaanxi Province, China (17). All individuals were healthy, mated naturally and kept under the same nutritional and living conditions. In addition, the litter size data for all goats were recorded by the stockmen.

The goat genomic DNA was extracted from tissue samples using a high-salt extraction protocol and then diluted to 10 ng/μL-concentration solution, keeping at −40°C (18, 19). The purity and quality of nucleic acid were detected using NanoDrop 1,000 instrument (Thermo Fisher Scientific Inc., America).

### 2.2 Plasmid construction

**Figure 1:**
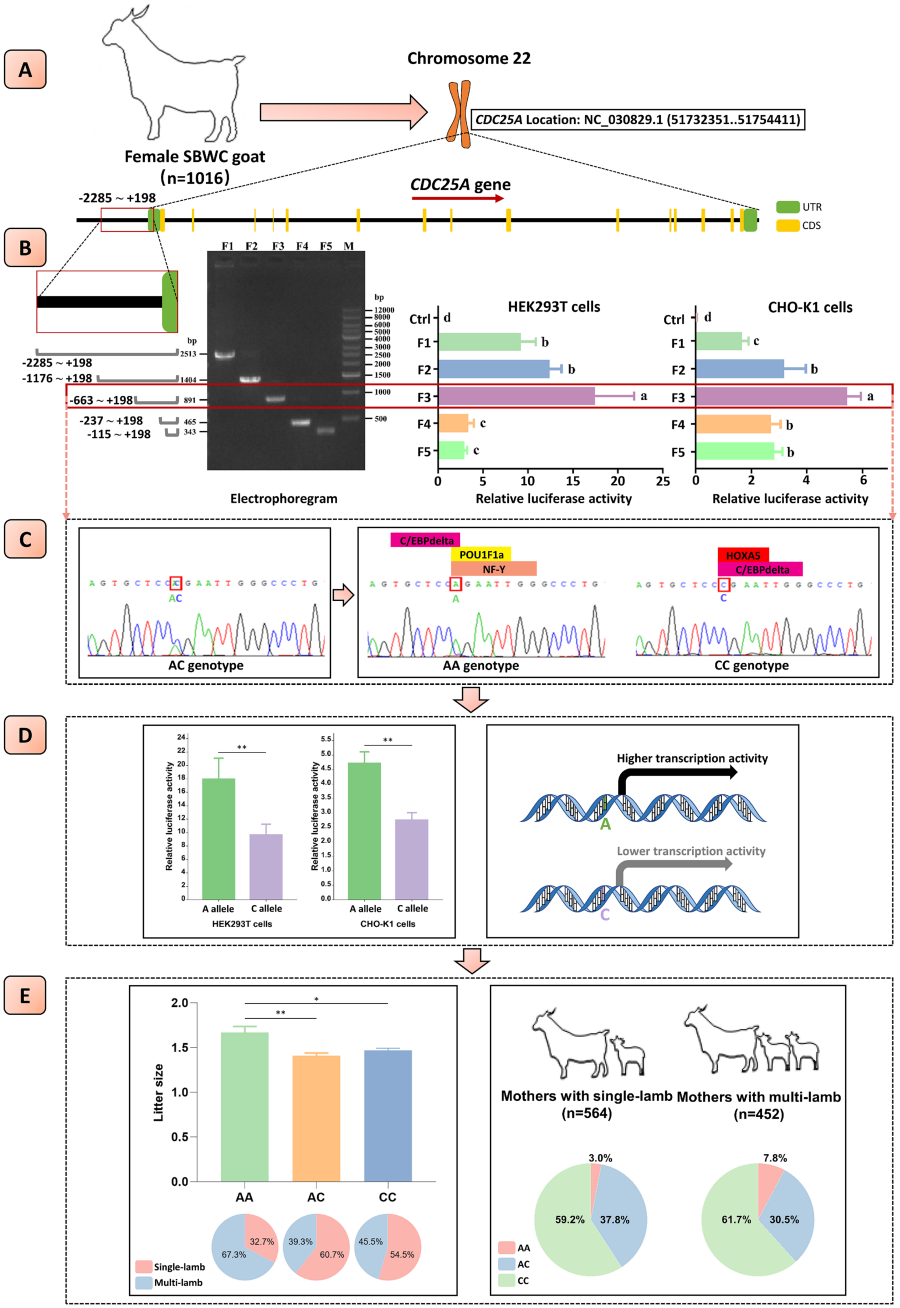
Mechanism diagram. **(A)** The structure patterns of goat *CDC25A* gene. **(B)** The core promoter of goat *CDC25A* gene was located at position-663 to-237. The electrophoregram on the left displayed that the five fragments were amplified successfully. The bar plots in the middle and on the right displayed the relative luciferase activity of different fragments within *CDC25A* gene promoter detected in HEK293T and CHO-K1 cells, respectively. **(C)** One novel SNP (NC_030829.1:g.51731829A > C) within the core promoter of goat *CDC25A* gene was found, which might change the binding of transcription factors. **(D)** The novel SNP significantly affected the transcriptional activity of *CDC25A* gene. **(E)** Left: associations between the novel SNP and litter size in goat *CDC25A* gene and the proportion of different types of litter size in individuals with AA, AC and CC genotypes. Right: the proportion of AA, AC and CC genotypes in single-lamb and multi-lamb groups. SBWC goat, Shaanbei white cashmere goat; UTR, untranslated region; CDS, coding sequence; **p < 0.01. Bars with different letters (a, b, c, d) means *p* < 0.05.

Five truncated fragments with different length divided in the-2285 nt ~ +198 nt region (F1: position-2285 nt to +198 nt; F2: position-1176 nt to +198 nt; F3: position-663 nt to +198 nt; F4: position-237 nt to +198 nt; F5: position-115 nt to +198 nt) were amplified and cloned into the pGL3-Basic luciferase reporter vectors using seamless cloning technology, respectively (Takara Bio, Kyoto, Japan) (Figure 1A, Information of primers was shown in Table 1). According to the target transferred fragments, plasmids were named as pGL3-F1, pGL3-F2, pGL3-F3, pGL3-F4 and pGL3-F5. Besides, the luciferase reporter vectors carrying various alleles were constructed according to the protocol described above after scanning the core promoter sequences. Recombinant vectors were used for transient transfection after identification. The pRL-TK vectors were used to normalize the luciferase signal, as the internal reference.

**Table 1:** Information of primers for goat *CDC25A* gene.

Primer name	Primer sequences (5′ → 3′)	Product length/bp	Utility
P1	F: ATCTGCGATCTAAGTGGCGAAAAACAGAGGTAGTGGT	2,513	F1 amplification and promoter activity detection
	R: GTAAGAGCTCGGTACCGGCAACCTTAAGATTAAATCCAAACA		
P2	F: TTTCTCTATCGATAGGACAAGTAATTTTCAGGACTGACAGCTG	1,404	F2 amplification and promoter activity detection
	R: GTAAGAGCTCGGTACCGGCAACCTTAAGATTAAATCCAAACA		
P3	F: TTTCTCTATCGATAGCGAGAGAAGGAAATGGTTCTGAG	891	F3 amplification and promoter activity detection
	R: GTAAGAGCTCGGTACCGGCAACCTTAAGATTAAATCCAAACA		
P4	F: TTTCTCTATCGATAGCAGCTGGGAGTTTTCATTGACC	465	F4 amplification and promoter activity detection
	R: GTAAGAGCTCGGTACCGGCAACCTTAAGATTAAATCCAAACA		
P5	F: CCGAGCTCTTACGCGATTGGTTCAGCCTAGCTGCC	343	F5 amplification and promoter activity detection
	R: GTAAGAGCTCGGTACCGGCAACCTTAAGATTAAATCCAAACA		
P6	F: AACGCAGGCCATAGGTTAAG	710	Mutation detection
	R: CAGGGGTCACACTCTCTTC		
	FAM:GAAGGTGACCAAGTTCATGCTACAGGAGCCTCAGTGCTCCA	68	KASP detection
P7	VIC: GAAGGTCGGAGTCAACGGATTACAGGAGCCTCAGTGCTCCC		
	R:TTGCCTCACAAACACAGGGC		

f, forward primer; r, reverse primer. The underline indicates the homology arms for seamless cloning. F1, fragment 1 (location-2285 ~ +198); F2, fragment 2 (location-1176 ~ +198); F3, fragment 3 (location-663 ~ +198); F4, fragment 4 (location-237 ~ +198); F5, fragment 5 (location-115 ~ +198).

### 2.3 Cell culture

Chinese hamster ovary K1 (CHO-K1) cells and Human embryonic kidney 293 T (HEK293T) cells were cultured in complete medium consisting of Ham’s F 12 nutrient medium (Sangon Biotech, Xi’an, China), 10% fetal bovine serum (Zeta life, America) and 1% penicillin–streptomycin (Gibco, America) at 37°C in a humidified 5% CO_2_ atmosphere (20–22).

### 2.4 Transient transfection and dual-luciferase reporter assay

Before transfection, the cells were seeded into 96-well plates at a density of 5 × 10^4^ cells per well. 100 μL of complete medium was added to each well and transient transfection was conducted after 12 h. 100 ng of reporter plasmids and 4 ng of transfection control plasmids (pRL-TK) to normalize the luciferase signal were co-transfected into CHO-K1 and HEK293T cells with Lipofectamine 2000 (Invitrogen, America). Then cells were cultured for 24 h and a Dual-Luciferase-Assay-System (Promega, Heidelberg, Germany) was employed to monitor luciferase activity (20–22).

### 2.5 Mutation detection and genotyping

A 25 μL polymerase chain reaction (PCR) system and the Kang’s program (21) were used to amplify the target fragments and products were validated by electrophoresis using 1.5% agarose gels stained with GoldView (Zhonghuihecai Biotech, Xi’an, China) (Information of primers was shown in Table 1 and the primer P6 was used to detect the novel mutation). Qualified PCR products were sent to sequencing to detect mutation located in the goat *CDC25A* core promoter (Sangon Biotech, Xi’an, China), which were then compared with variant tables of Ensembl Database^2^ and Animal Omics Database (AOD)^3^ to search novel nucleotide polymorphisms.

Besides, genotyping was performed with the KASP™ genotyping technology (Kompetitive Allele-Specific PCR assay, FLU-ARMS, Videgene, China), and results were visualized with fluorescence ration PCR instrument (Quant Studio 5, Thermo Fisher Scientific, US). Genotype data for each animal were exported for the statistical analysis.

### 2.6 Statistical analyses

The frequencies of genotypes and alleles, heterozygosity (He), homozygosity (Ho), and polymorphism information content (PIC) of the mutation were calculated adopting Nei’s methods (23). Welch’s ANOVA, *Post Hoc* Multiple Comparisons (Tamhane’s T2 test) and *χ^2^* test were used to analyze the correlation between the mutation and litter size. Hardy–Weinberg equilibrium was tested in SHEsis software^4^ (24). The following general linear model of ANOVA was used: Y*_ijk_* = *μ* + A*_i_* + G*_j_* + ε*_ijk_*, where Y*_ijk_* is the phenotypic data of litter size of each animal, μ is the mean of population, A*_i_* is the fixed effect of age, G*_j_* is the fixed effect of genotype, and ε*_ijk_* is the random error (25).

### 2.7 Prediction of CpG island and transcription factors

Promoter regions often contains CpG islands (26), which have been linked to promoter activity (27). DNA methylation at CpG islands is crucial for gene transcription and tissue-specific processes (28). Thus, we predicted the CpG islands in the *CDC25A* promoter using the EMBOSS Cpgplot software.^5^ Besides, a mutation in the core promoter may affect transcriptional activity by changing transcription factors bound with core promoters (29), and thus potential transcription factors were predicted via the PROMO software^6^ (factors predicted within a dissimilarity margin less or equal than 5%).

## 3 Results

### 3.1 Identification of the goat *CDC25A* gene core promoter

Five truncated *CDC25A* promoter fragments were successfully amplified (Figure 1B) and cloned into luciferase reporter vectors, which were co-transfected with pRL-TK into CHO-K1 and HEK293T cells, respectively. The dual-luciferase reporter assay displayed that the pGL3-F3 vector had significantly stronger luminescence activity than the others in both cell lines (*p* < 0.05) (Figure 1B), suggesting that the core promoter was located at position-663 nt to-237 nt of *CDC25A* gene.

### 3.2 Detection and genetic parameter analysis of a novel SNP within the goat *CDC25A* core promoter

The core promoter sequences were amplified and sent to sequencing in order to scan the nucleotide polymorphisms among a population of 413 goats. One SNP (NC_030829.1:g.51731829A > C) within the core promoter was identified and three genotypes, AA, AC and CC, were detected, which had never been recorded in the Ensembl Database and AOD (Figure 1C). Then, the nucleotide diversity of the remaining 603 individuals was detected by KASP technique (Supplementary Figure S2). The frequencies of AA, AC and CC were 0.051, 0.346 and 0.603, and the A and C allele frequencies were 0.224 and 0.776, respectively. The genetic parameters were calculated as follows: *Ho* = 0.652, *He* = 0.348, *Ne* = 1.533, and *PIC* = 0.287 (Table 2). These observations indicated that the frequency of the novel SNP was high, and it had a moderate level of genetic diversity (0.25 < *PIC* < 0.5). Besides, the mutation is at the Hardy–Weinberg equilibrium in this population (*p* = 0.5318) (Table 2).

**Table 2:** Genetic parameters of the SNP (NC_030829.1:g.51731829A > C) within *CDC25A* in SBWC goats (*n* = 1,016).

Genotypic frequencies			Allelic frequencies		Population parameters				*H*
AA	AC	CC	A	C	*Ho*	*He*	*Ne*	*PIC*	
0.051 (*n* = 52)	0.346 (*n* = 351)	0.603 (*n* = 613)	0.224	0.776	0.652	0.348	1.533	0.287	0.5318

Ho, homozygosity; He, heterozygosity; Ne, Number of effective alleles; PIC, polymorphism information content.

### 3.3 The novel SNP might change the binding of transcription factors

In this study, the CpG islands in promoter and transcription factors bound with the promoter have been predicted. A same CpG island, located at position +96 to +445, and 350 bp in length, was predicted in both alleles and 43% length of the CpG island region was coincident with the core promoter using the online software EMBOSS Cpgplot (Supplementary Figure S4). However, the transcription factors binding at the SNP locus varied between different alleles. Homeobox A5 (HOXA5) was observed to bind to the C allele, and POU Class 1 Homeobox 1 (POU1F1/POU1F1a) and Nuclear Transcription Factor Y (NF-Y) were observed to bind to the A allele.

### 3.4 The novel SNP significantly affected the transcriptional activity of *CDC25A*

The core promoter can regulate gene expression activity via binding transcription factors. Consequently, a mutation in the core promoter can alter transcriptional activity and then affect phenotypic traits in animals. In order to confirm whether the SNP would affect the transcriptional activity of the *CDC25A* gene, luciferase reporter vectors were constructed. The results demonstrated that the luminescence activity of vectors carrying the A allele was significantly higher than vectors carrying the C allele in both CHO-K1 and HEK293T cells (Figure 1D).

### 3.5 Correlation between the novel SNP and goat litter size

We examined the relationship between the novel SNP and litter size using Welch’s ANOVA, followed by Tamhane’s T2 *post hoc* test for multiple comparisons to assess the significance of litter size differences across genotypes. Additionally, the *χ^2^* test was applied to evaluate differences in genotype distribution among goats with varying litter sizes in the population. The association analysis revealed a significant correlation between the novel SNP and litter size in goats. Individuals with the AA genotype had a higher litter size compared to those with the CC and AC genotypes (*p* < 0.05). Moreover, goats with the AA genotype were more prevalent among mothers with multiple lambs than among those with single lambs (*p* < 0.05) (Table 3, Figure 1E).

**Table 3:** Genotype distribution between mothers with single and multi-lamb in SBWC goats.

Types	Sample sizes	Genotypes			Independent *χ^2^*, (*p* value)
		AA	AC	CC	
Mothers with single-lamb	564	17	213	334	*χ^2^* = 15.027, (*p* = 0.001)
Mothers with multi-lamb	452	35	138	279	

## 4 Discussion

MAS, a modern approach within the field of molecular breeding (30), offers the advantages of high efficiency and precision. It has been applied to enhance reproductive traits and overall productivity in goats (17). The *CDC25A* gene has been demonstrated to be essential for spermatogenesis and oocyte development (5–7, 31) and selected as a candidate gene for goat breeding (2, 10–12, 32). Previous research has demonstrated that mutations in the coding regions and introns of the *CDC25A* gene influence litter size in goats. However, there have been limited studies examining the relationship between promoter polymorphisms and reproductive traits in goats.

We first investigated the regional coverage of the *CDC25A* core promoter. Five truncated fragments with different length located from position −2285 to +198 were amplified and linked to pGL3-Basic plasmids. A dual-luciferase reporter assay was used to detect luminescence activity and the CHO-K1 and HEK293T cell lines were implied for transfection. The results displayed that maximum luminescence activity was observed at the PGL3-F3 vector in both cell lines, suggesting the core promoter of *CDC25A* was located at position-663 to-237 (Figure 1B).

Mutations in gene promoters were demonstrated to alter animal phenotypic traits, including litter size, milk traits, and body measurement traits, etc. (21, 33, 34). In our study, one novel SNP (NC_030829.1:g.51731829A > C) located in the *CDC25A* core promoter was successfully detected among 1,016 goats (Figure 1B). Genetic parameter analysis demonstrated that the frequency of the C allele was higher than the A allele in SBWC breed. Besides, the SNP had a moderate level of genetic diversity and at the Hardy–Weinberg equilibrium (*p* = 0.5318) in this population (Table 2) (17).

A mutation within the core promoter can impact epigenetic processes and alter transcription factor binding. Thus, CpG islands in the promoter and transcription factors bound to the SNP locus were predicted. We found that the mutation did not affect the existence of CpG island but changed the binding of the transcription factors (Supplementary Figure S4). Notably, POU1F1a, a key transcription factor that influences development and growth through direct regulation of pituitary hormone secretion (35), could combine with the A allele rather than the C allele (Figure 1C). The missense mutation within *POU1F1a* has been reported to be strongly related to litter size in goat (25), suggesting that the novel SNP might cause binding changes of important transcription factors and affect growth or reproduction phenotypes in goat.

The changes in transcription factor binding can alter the transcriptional activity of genes (36). Therefore, to investigate whether the SNP influences gene transcriptional activity, vectors containing the A allele and C allele were constructed. The results revealed that the vector with the A allele exhibited significantly higher luminescence activity, confirming that the SNP in the CDC25A core promoter affects the gene’s transcriptional activity (Figure 1D). Notably, the up or down-regulation of the transcriptional activity of genes could alter phenotypic traits of animals. We found that the litter size of goats with the AA genotype was significantly better than those with the AC or CC genotype, which corresponded to increased transcriptional activity in plasmids carrying the A allele, and amounts of mothers with single/multi-lamb were significantly correlated with genotype frequencies. Goats with the AA genotype accounted for a higher proportion in the multi-lamb population compared with the single-lamb population. It has been indicated that *CDC25A* activity is crucial for both spermatogenesis (5, 6, 31) and oocyte development, particularly during the metaphase II arrest in oocytes (7). Therefore, the SNP may affect the expression of the *CDC25A* gene, thereby influencing downstream physiological processes, particularly reproductive performance. In summary, the novel SNP within the core promoter of the *CDC25A* gene changed promoter activity and was remarkably associated with litter size in goat.

Our findings would provide a potential molecular genetic marker for improving goat reproductive efficiency. However, more evidences are required to determine whether the correlation between the novel SNP and litter size is direct or indirect.

## 5 Conclusion

In the present study, the core promoter of the goat *CDC25A* gene was identified and a novel SNP in the core promoter was associated with the litter size presumably by affecting transcriptional activity. The novel SNP locus could be a potential molecular genetic marker to improve goat reproductive efficiency.

## Data Availability

The variant data for this study have been deposited in the European Variation Archive (EVA) at EMBL-EBI under accession number PRJEB84361, https://www.ebi.ac.uk/eva/?eva-study=PRJEB84361.

## Footnotes

^1^ http://animal.nwsuaf.edu.cn/code/index.php/RGD

^2^ http://asia.ensembl.org/index.html

^3^ http://animal.nwsuaf.edu.cn/

^4^ http://analysis.bio-x.cn

^5^ https://www.ebi.ac.uk/Tools/seqstats/emboss_cpgplot/

^6^ http://alggen.lsi.upc.es/cgi-bin/promo_v3/promo/promoinit.cgi?dirDB=TF_8.3
