# Supplementary material for: A functional SNP of the core promoter region within goat CDC25A gene affects litter size
Source: Front Vet Sci. 2025 Feb 5;11:1471123. doi: 10.3389/fvets.2024.1471123 (PMC11841496; doi:10.3389/fvets.2024.1471123)
Supplement: Supplementary file 1 [file Data_Sheet_1.docx]

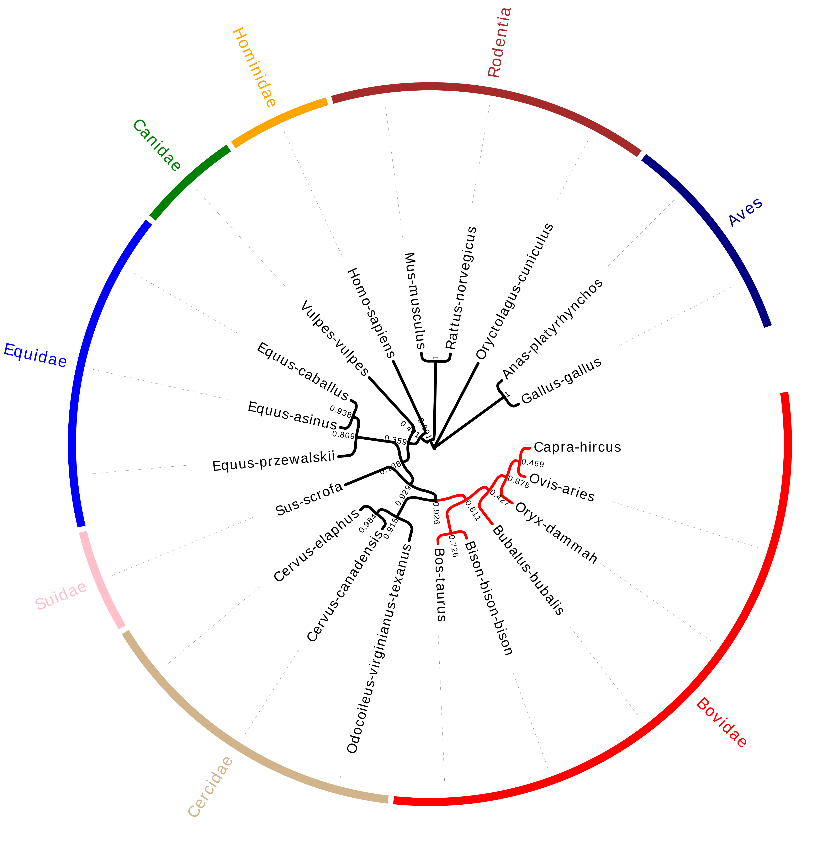


**Figure S1.** Phylogenetic trees of *CDC25A* gene among common animals. Branches of *Bovidae* including goat (*Capra hirus*) were highlighted with red.


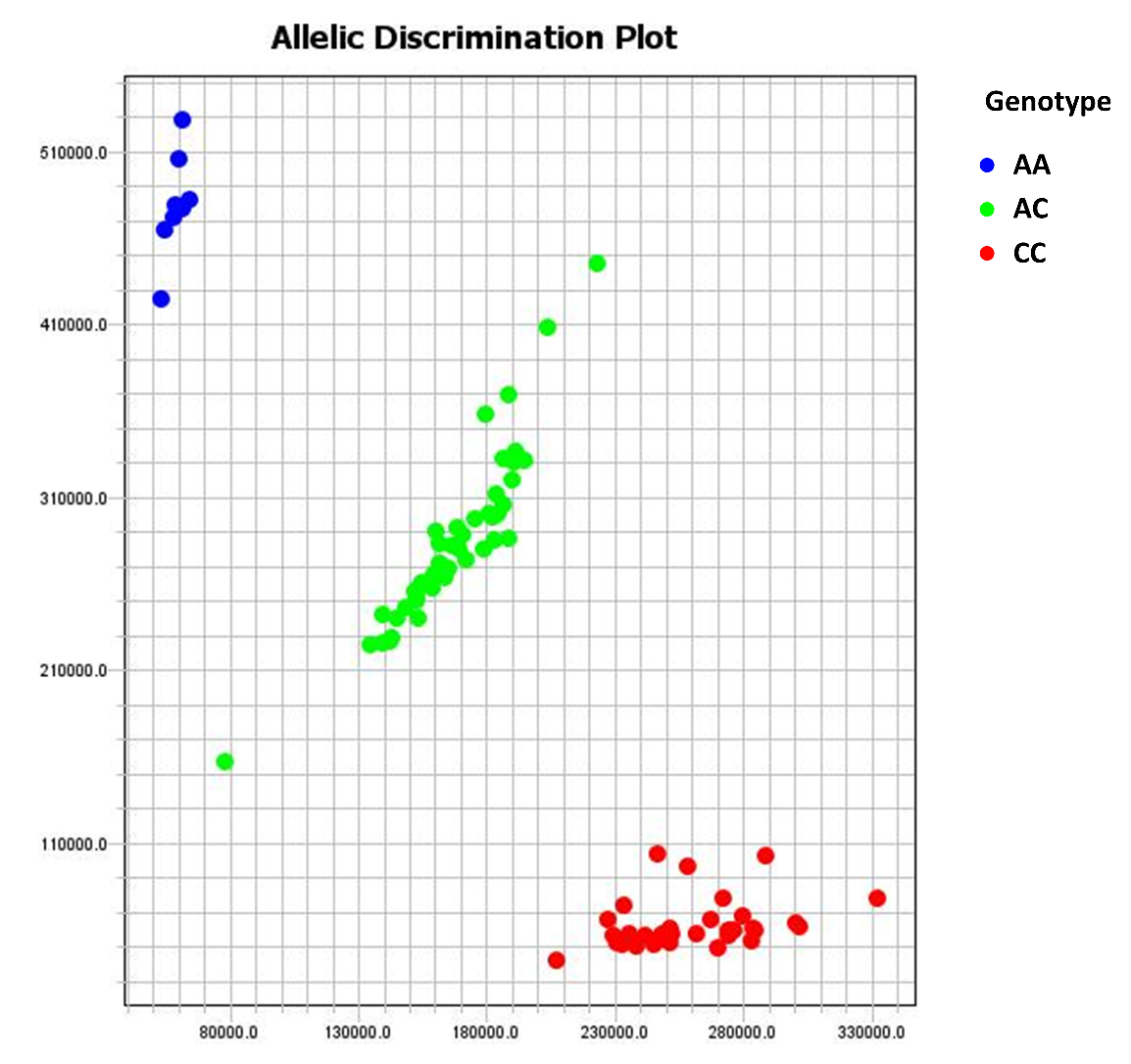


**Figure S2.** The KASP genotyping results for the SNP in SBWC goat.


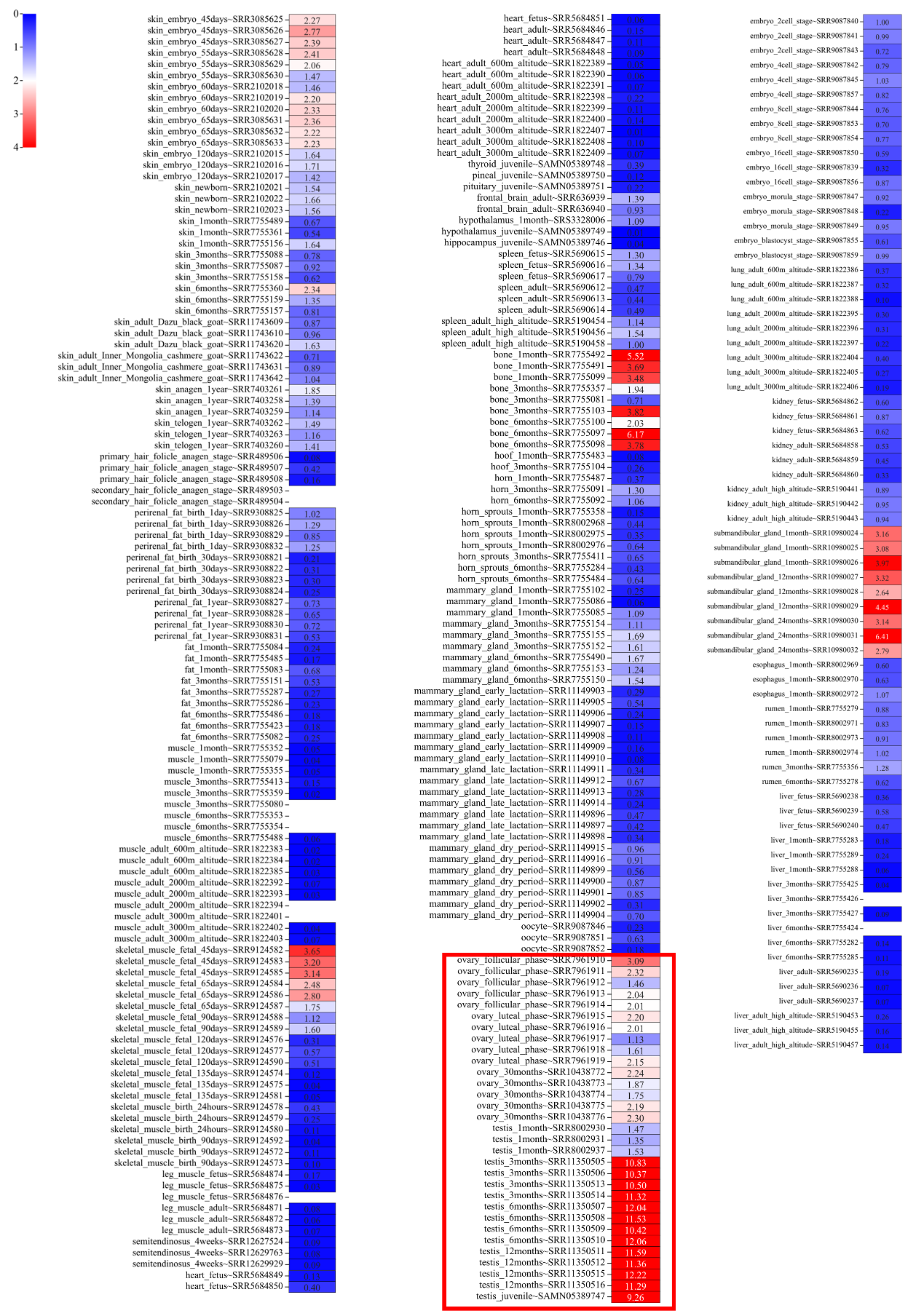


**Figure S3.** Heat map of expression analysis (FPKM) of goat *CDC25A* gene in different tissues. The expression levels of testis and ovary were marked by the red box.


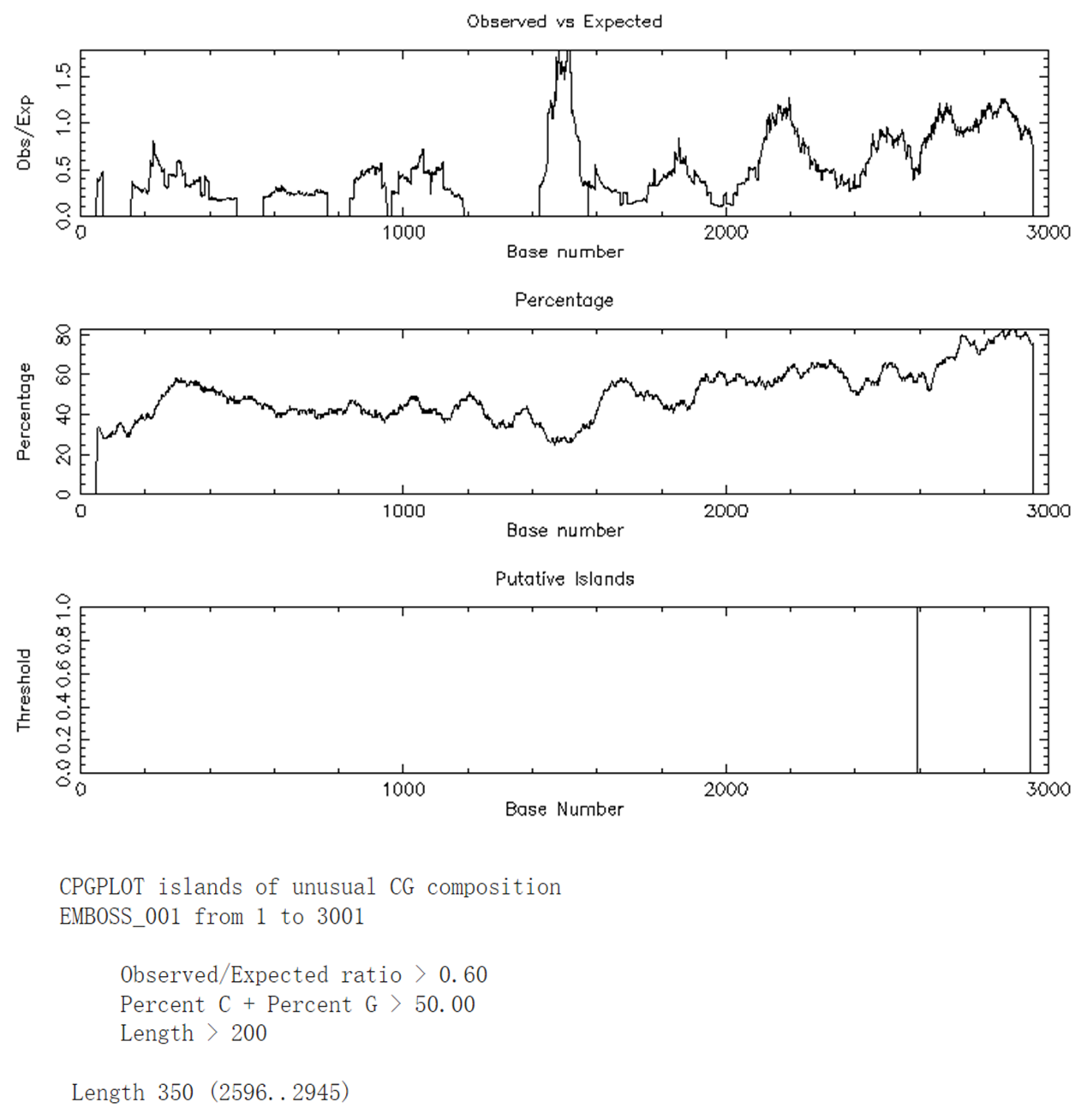


**Figure S4.** Prediction of CpG islands in promoter of goat *CDC25A* gene.
